# Crystal structure of 1,1′-[imidazolidine-1,3-diylbis(methyl­ene)]bis­(naphthalen-2-ol)

**DOI:** 10.1107/S2056989015002078

**Published:** 2015-02-07

**Authors:** Augusto Rivera, Jicli José Rojas, Jaime Ríos-Motta, Michael Bolte

**Affiliations:** a Departamento de Química, Facultad de Ciencias, Universidad Nacional de Colombia, Sede Bogotá, Cra 30 No. 45-03, Bogotá, Colombia; b Institut für Anorganische Chemie, Goethe-Universität, Max-von-Laue-Strasse 7, Frankfurt/Main D-60438, Germany

**Keywords:** crystal structure, imadazolidine, (2-hy­droxy­naphthalen-1-yl)meth­yl, hydrogen bonding

## Abstract

The crystal structure of the title compound, C_25_H_24_N_2_O_2_, at 173 K has monoclinic (*C*2/*c*) symmetry. The mol­ecule is located on a crystallographic twofold rotation axis with only half a mol­ecule in the asymmetric unit. The imidazolidine ring adopts a twist conformation, with a twist about the ring C—C bond. The crystal structure shows the *anti­clinal* disposition of the two (2-hy­droxy­naphthalen-1-yl)methyl substituents of the imidazolidine ring. The structure displays two intra­molecular O—H⋯N hydrogen bonds, each forming an *S*(6) ring motif.

## Abstract 2

The crystal structure of the title compound displays a twist conformation of the imidazolidine ring with two (2-hy­droxy­naphthalen-1-yl)methyl substituents stabilized by two intra­molecular O—H⋯N hydrogen bonds.

## Chemical context   

We have been inter­ested in the synthesis and characterization of a family of symmetrical *N*,*N*′-disubstituted imidazolidines by the use of a Mannich-type condensation of *cyclic cage aminals* with phenols in a one-pot reaction. The main structural feature of the symmetrical *N*,*N*′-disubstituted imidazolidines, the so-called aromatic *di*-Mannich bases, is to form intra­molecular hydrogen bonds that reveal great structural and thermodynamic stability. These *di*-Mannich bases which contain a phenolic or naphtho­lic hydroxyl group as a proton donor, as well as an *ortho*-amino­methyl group as a proton acceptor in the same mol­ecule are convenient models for studying the nature of hydrogen bonding and other weak non-covalent inter­actions (Koll *et al.*, 2006 ▸).
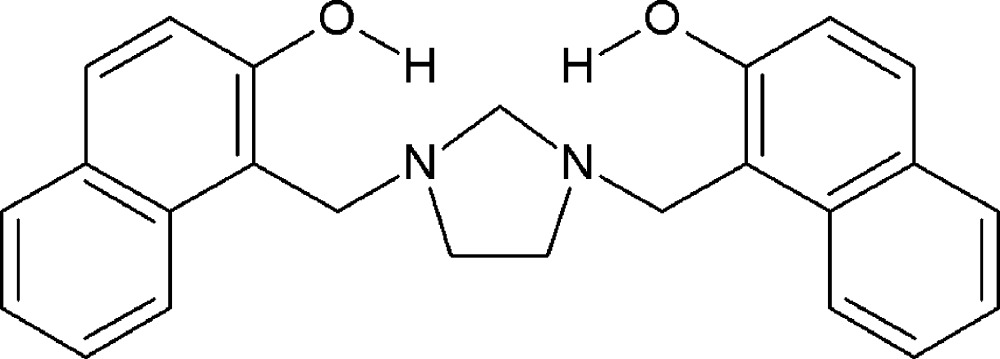


 In previous studies (Rivera *et al.*, 2006 ▸), 1,1′-[imidazolidine-1,3-diylbis(methyl­ene)]bis­(naphthalen-2-ol), (I), was obtained in good yields by an one-pot Mannich-type reaction involving 1,3,6,8-tetra­aza­tri­cyclo­[4.4.1.1^3,8^]dodecane (TATD) and naph­thalen-2-ol in classical solvents for Mannich reactions, such as dioxane or ethanol. Intriguingly, reactions of 1,3,6,8-tetra­aza­tri­cyclo­[4.4.1.1^3,8^]dodecane (TATD) with naphthalen-2-ol may lead to other results. It has been found (Rivera & Quevedo, 2013 ▸) that inter­action of TATD with naphthalen-2-ol in solvent-free conditions by heating in an oil bath a 1:4 mixture with stirring at 423 K for 20 min gives 1,1′-methyl­enebis(naphthalen-2-ol) in good yields. On the other hand, the reactions of TATD with naphthalen-2-ol under solvent-free microwave-assisted conditions yields the title compound and no formation of 1,1′-methyl­enebis(naphthalen-2-ol) was observed. In contrast to classical Mannich reaction conditions this reaction required neither solvent nor inert atmosphere conditions.

## Structural commentary   

**Figure 1:**
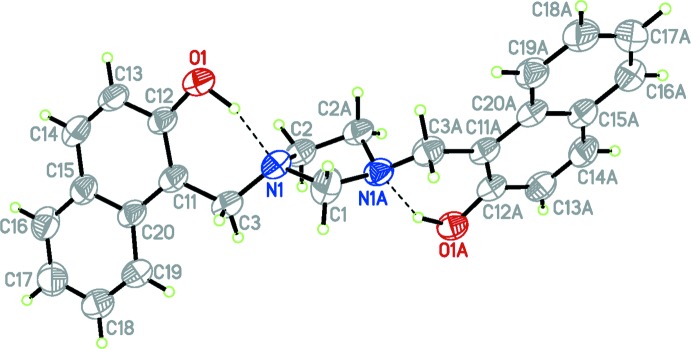
The mol­ecular structure of the title compound, with displacement ellipsoids drawn at the 50% probability level. Hydrogen bonds are drawn as dashed lines. Atoms labelled with the suffix ‘A’ are generated using the symmetry operator (−*x* + 1, *y*, −*z* + 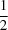).

In contrast to the closely related structure (Rivera *et al.*, 2012*a* ▸), which crystallized in the monoclinic *P*2_1_/*n* space group, the title compound crystallizes in the *C*2/*c* space group. The mol­ecular structure is shown in Fig. 1 ▸. The asymmetric unit contains one half mol­ecule and the whole mol­ecule is generated by twofold rotational symmetry (see Fig. 1 ▸). The near planarity of the fused aromatic ring system is illustrated by the very small deviation of all the atoms from the plane [largest deviation = 0.0227 (17) Å for atom C11]. The imidazolidine ring (C1/N1/C2/C2′/N1′) is in a twisted conformation on C2—C2′, with puckering parameters *Q*(2) = 0.4126 (17) Å and ϕ(2) = 126.0 (2)° (Cremer & Pople, 1975 ▸). The crystal structure shows the *anti­clinal* disposition of the two (2-hy­droxy­naphthalen-1-yl)methyl substituents of the imidazolidine ring [pseudo-torsion angle CH_2_—N⋯N—CH_2_ = −121.77 (18)°]. The mean plane of the imidazolidine ring, defined by atoms N1, C1 and N1′, makes a dihedral angle of 70.92 (4)° with the pendant aromatic rings (C11–C20). The dihedral angle between the planes of the naphthyl rings is 60.55 (4)°.

As with related structures in this series, the mol­ecular conformation is stabilized by two intra­molecular O—H⋯N hydrogen-bond inter­actions with *S*(6) graph-set motifs (Bernstein *et al.*, 1995 ▸). Due to symmetry and contrary to other structures, where hydrogen-bond distances were different, the two observed intra­molecular hydrogen-bond distances were identical (Table 1 ▸).

## Supra­molecular features   

**Figure 2:**
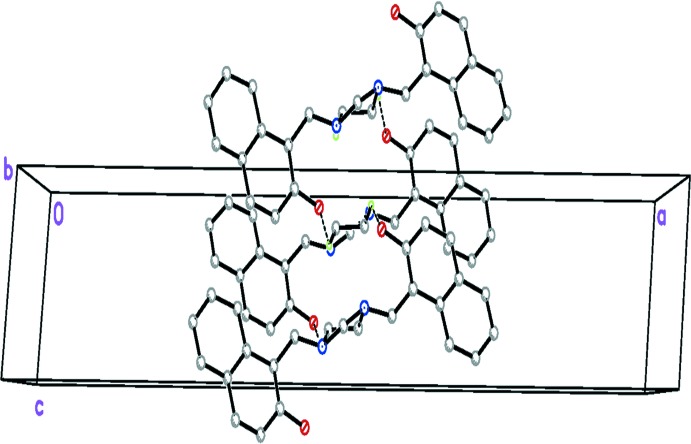
The crystal packing of the title compound, howing one of the zigzag chains that extend along the crystal *c*-axis direction. Hydrogen bonds are drawn as dashed lines.

Unlike the situation found in related structures, there is only one significant inter­molecular inter­action involving the O—H group (as acceptor) and a methyl­ene-H atom (as donor) to consolidate the crystal packing. These weak inter­actions led to the formation of parallel sets of zigzag chains extending along the *c* axis of the crystal (Fig. 2 ▸).

## Database survey   

A search in the Cambridge Structural Database (Groom & Allen, 2014 ▸) for the fragment 2,2′-[imidazolidine-1,3-diylbis(methyl­ene)]diphenol yielded seven hits, namely 2,2′-[imidazolidine-1,3-diylbis(methyl­ene)]bis­(4-*tert*-butyl­phenol) (Rivera, Nerio & Bolte, 2013 ▸), 2,2′-[imidazolidine-1,3-diyl­bis(methyl­ene)]bis­(4-chloro­phenol) (Rivera *et al.*, 2011 ▸), 2,2′-[imidazolidine-1,3-diylbis(methyl­ene)]bis­[4-(2,4,4-tri­methyl­pen­tan-2-yl)phenol] (Kober *et al.*, 2012 ▸), 4,4′-di­fluoro-2,2′-[imidazolidine-1,3-diylbis(methyl­ene)]diphenol (Rivera *et al.*, 2012*b* ▸) 2,2′-[imidazolidine-1,3-diylbis(methyl­ene)]bis­(6-methyl­phenol) (Rivera *et al.*, 2014 ▸), 2,2′-[imidazolidine-1,3-diyl­bis(methyl­ene)]diphenol (Rivera *et al.*, 2012*b* ▸) and 4,4′-di­methyl-2,2′-[imidazolidine-1,3-diylbis(methyl­ene)]diphenol (Rivera *et al.*, 2012*c* ▸). In all of these compounds, the hy­droxy groups in the *ortho* position of the aromatic ring form an intra­molecular hydrogen bond to an N atom of the imidazoline ring.

## Synthesis and crystallization   

The title compound has been synthesized in solution according to a literature procedure (Rivera *et al.*, 2006 ▸); however, in this instance, the synthesis was carried out under microwave-assisted solvent free conditions. A mixture of 1 mmol of 1,3,6,8-tetra­aza­tri­cyclo­[4.4.1.1^3,8^]dodecane (TATD) and 2 mmol of naphthalen-2-ol was subjected to microwave irradiation (200 W) for 10 min at a temperature of 373 K. The product was washed with water and then with benzene (yield 94%, m.p. 435–436 K). Crystals suitable for X-ray diffraction were obtained from a methanol solution upon slow evaporation of the solvent at room temperature.

## Refinement details   

Crystal data, data collection and structure refinement details are summarized in Table 2 ▸. All H atoms were located in the difference electron-density map. The hy­droxy H atom was refined freely, while C-bound H atoms were fixed geometrically (C—H = 0.95 or 0.99 Å) and refined using a riding model, with *U*_iso_(H) values set at 1.2*U*_eq_ of the parent atom.

## Supplementary Material

Crystal structure: contains datablock(s) I, New_Global_Publ_Block. DOI: 10.1107/S2056989015002078/sj5441sup1.cif

Structure factors: contains datablock(s) I. DOI: 10.1107/S2056989015002078/sj5441Isup2.hkl

Supporting information file. DOI: 10.1107/S2056989015002078/sj5441Isup3.cml

CCDC reference: 1046536

Additional supporting information:  crystallographic information; 3D view; checkCIF report

## Figures and Tables

**Table 1:** Hydrogen-bond geometry (, )

*D*H*A*	*D*H	H*A*	*D* *A*	*D*H*A*
O1H1N1	1.05(2)	1.65(2)	2.6143(19)	151.0(19)
C2H2*A*O1^i^	0.99	2.64	3.257(2)	121

Symmetry code: (i) 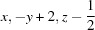.

**Table 2:** Experimental details

Crystal data	
Chemical formula	C_25_H_24_N_2_O_2_
*M*_r_	384.46
Crystal system, space group	Monoclinic, *C*2/*c*
Temperature (K)	173
*a*, *b*, *c* ()	34.883(5), 8.3956(9), 6.5830(8)
()	95.650(11)
*V* (^3^)	1918.6(4)
*Z*	4
Radiation type	Mo *K*
(mm^1^)	0.09
Crystal size (mm)	0.19 0.17 0.11

Data collection	
Diffractometer	Stoe IPDS II two circle
Absorption correction	Multi-scan (*X-AREA*; Stoe Cie, 2001 ▸)
*T*_min_, *T*_max_	0.972, 0.989
No. of measured, independent and observed [*I* > 2(*I*)] reflections	8297, 1852, 1451
*R*_int_	0.090
(sin /)_max_ (^1^)	0.616

Refinement	
*R*[*F* ^2^ > 2(*F* ^2^)], *wR*(*F* ^2^), *S*	0.055, 0.159, 1.09
No. of reflections	1852
No. of parameters	136
H-atom treatment	H atoms treated by a mixture of independent and constrained refinement
_max_, _min_ (e ^3^)	0.24, 0.23

Computer programs: *X-AREA* (Stoe Cie, 2001 ▸), *SHELXS97* and *XP* (Sheldrick, 2008 ▸), *SHELXL2014* (Sheldrick, 2015 ▸) and *publCIF* (Westrip, 2010 ▸).

## supplementary crystallographic information

### Crystal data

**Table:**

C_25_H_24_N_2_O_2_	*F*(000) = 816
*M**_r_* = 384.46	*D*_x_ = 1.331 Mg m^−^^3^
Monoclinic, *C*2/*c*	Mo *K*α radiation, λ = 0.71073 Å
*a* = 34.883 (5) Å	Cell parameters from 8026 reflections
*b* = 8.3956 (9) Å	θ = 2.4–26.2°
*c* = 6.5830 (8) Å	µ = 0.09 mm^−^^1^
β = 95.650 (11)°	*T* = 173 K
*V* = 1918.6 (4) Å^3^	Block, colourless
*Z* = 4	0.19 × 0.17 × 0.11 mm

### Data collection

**Table:**

Stoe IPDS II two-circle diffractometer	1451 reflections with *I* > 2σ(*I*)
ω scans	*R*_int_ = 0.090
Absorption correction: multi-scan *X-AREA* (Stoe & Cie, 2001)	θ_max_ = 26.0°, θ_min_ = 2.5°
*T*_min_ = 0.972, *T*_max_ = 0.989	*h* = −42→34
8297 measured reflections	*k* = −10→10
1852 independent reflections	*l* = −8→8

### Refinement

**Table:**

Refinement on *F*^2^	0 restraints
Least-squares matrix: full	Hydrogen site location: mixed
*R*[*F*^2^ > 2σ(*F*^2^)] = 0.055	H atoms treated by a mixture of independent and constrained refinement
*wR*(*F*^2^) = 0.159	*w* = 1/[σ^2^(*F*_o_^2^) + (0.085*P*)^2^ + 0.4089*P*] where *P* = (*F*_o_^2^ + 2*F*_c_^2^)/3
*S* = 1.09	(Δ/σ)_max_ < 0.001
1852 reflections	Δρ_max_ = 0.24 e Å^−^^3^
136 parameters	Δρ_min_ = −0.23 e Å^−^^3^

### Special details

**Table:**

Geometry. All e.s.d.'s (except the e.s.d. in the dihedral angle between two l.s. planes) are estimated using the full covariance matrix. The cell e.s.d.'s are taken into account individually in the estimation of e.s.d.'s in distances, angles and torsion angles; correlations between e.s.d.'s in cell parameters are only used when they are defined by crystal symmetry. An approximate (isotropic) treatment of cell e.s.d.'s is used for estimating e.s.d.'s involving l.s. planes.

### Fractional atomic coordinates and isotropic or equivalent isotropic displacement parameters (Å^2^)

**Table:**

	*x*	*y*	*z*	*U*_iso_*/*U*_eq_	Occ. (<1)
O1	0.45368 (4)	0.77294 (16)	0.70176 (19)	0.0507 (4)	
H1	0.4677 (6)	0.723 (3)	0.583 (4)	0.061 (6)*	
N1	0.47179 (4)	0.69601 (18)	0.3384 (2)	0.0455 (4)	
C1	0.5000	0.5948 (4)	0.2500	0.0627 (8)	
H1A	0.5132	0.5258	0.3570	0.075*	0.5
H1B	0.4868	0.5258	0.1430	0.075*	0.5
C2	0.47856 (5)	0.8543 (2)	0.2548 (2)	0.0488 (5)	
H2A	0.4707	0.9396	0.3459	0.059*	
H2B	0.4646	0.8678	0.1175	0.059*	
C3	0.43203 (5)	0.6374 (2)	0.2997 (3)	0.0491 (5)	
H3A	0.4319	0.5212	0.3249	0.059*	
H3B	0.4231	0.6549	0.1540	0.059*	
C11	0.40403 (5)	0.7157 (2)	0.4287 (2)	0.0437 (4)	
C12	0.41619 (5)	0.7796 (2)	0.6188 (2)	0.0446 (4)	
C13	0.38994 (6)	0.8541 (2)	0.7379 (3)	0.0511 (5)	
H13	0.3990	0.8993	0.8660	0.061*	
C14	0.35197 (6)	0.8623 (2)	0.6724 (3)	0.0541 (5)	
H14	0.3348	0.9149	0.7536	0.065*	
C15	0.33758 (5)	0.7931 (2)	0.4833 (3)	0.0499 (5)	
C16	0.29795 (6)	0.7964 (3)	0.4150 (3)	0.0611 (6)	
H16	0.2805	0.8489	0.4948	0.073*	
C17	0.28426 (6)	0.7252 (3)	0.2355 (3)	0.0682 (6)	
H17	0.2575	0.7286	0.1910	0.082*	
C18	0.30975 (6)	0.6476 (3)	0.1178 (3)	0.0662 (6)	
H18	0.3001	0.5965	−0.0055	0.079*	
C19	0.34828 (6)	0.6438 (3)	0.1770 (3)	0.0545 (5)	
H19	0.3650	0.5908	0.0938	0.065*	
C20	0.36387 (5)	0.7181 (2)	0.3618 (2)	0.0455 (5)	

### Atomic displacement parameters (Å^2^)

**Table:**

	*U*^11^	*U*^22^	*U*^33^	*U*^12^	*U*^13^	*U*^23^
O1	0.0588 (8)	0.0562 (8)	0.0367 (6)	−0.0007 (6)	0.0024 (5)	−0.0039 (5)
N1	0.0551 (9)	0.0469 (8)	0.0350 (7)	0.0057 (6)	0.0072 (6)	−0.0001 (6)
C1	0.0688 (18)	0.0559 (17)	0.0670 (17)	0.000	0.0256 (14)	0.000
C2	0.0617 (11)	0.0505 (11)	0.0342 (8)	0.0027 (8)	0.0047 (7)	0.0023 (7)
C3	0.0615 (12)	0.0496 (10)	0.0364 (8)	−0.0001 (8)	0.0061 (7)	−0.0058 (7)
C11	0.0590 (11)	0.0412 (9)	0.0318 (8)	0.0009 (8)	0.0086 (7)	0.0016 (6)
C12	0.0574 (11)	0.0438 (10)	0.0331 (8)	−0.0026 (7)	0.0067 (7)	0.0024 (6)
C13	0.0676 (12)	0.0525 (11)	0.0342 (8)	−0.0042 (9)	0.0098 (8)	−0.0063 (7)
C14	0.0667 (12)	0.0548 (11)	0.0436 (9)	0.0030 (9)	0.0192 (8)	−0.0039 (8)
C15	0.0570 (11)	0.0539 (11)	0.0399 (9)	−0.0012 (8)	0.0107 (7)	0.0054 (7)
C16	0.0597 (12)	0.0746 (14)	0.0508 (11)	0.0032 (10)	0.0141 (9)	0.0079 (9)
C17	0.0549 (12)	0.0951 (18)	0.0539 (12)	−0.0018 (11)	0.0020 (9)	0.0091 (11)
C18	0.0678 (14)	0.0890 (16)	0.0409 (10)	−0.0065 (12)	0.0010 (9)	0.0002 (10)
C19	0.0614 (12)	0.0646 (12)	0.0376 (9)	−0.0022 (9)	0.0055 (8)	−0.0020 (8)
C20	0.0602 (11)	0.0455 (10)	0.0316 (8)	−0.0021 (8)	0.0082 (7)	0.0036 (6)

### Geometric parameters (Å, º)

**Table:**

O1—C12	1.368 (2)	C12—C13	1.409 (3)
O1—H1	1.05 (2)	C13—C14	1.354 (3)
N1—C1	1.464 (2)	C13—H13	0.9500
N1—C2	1.467 (2)	C14—C15	1.420 (3)
N1—C3	1.470 (2)	C14—H14	0.9500
C1—N1^i^	1.464 (2)	C15—C16	1.411 (3)
C1—H1A	0.9900	C15—C20	1.422 (3)
C1—H1B	0.9900	C16—C17	1.368 (3)
C2—C2^i^	1.503 (4)	C16—H16	0.9500
C2—H2A	0.9900	C17—C18	1.397 (3)
C2—H2B	0.9900	C17—H17	0.9500
C3—C11	1.507 (2)	C18—C19	1.362 (3)
C3—H3A	0.9900	C18—H18	0.9500
C3—H3B	0.9900	C19—C20	1.427 (2)
C11—C12	1.389 (2)	C19—H19	0.9500
C11—C20	1.427 (3)		
			
C12—O1—H1	102.4 (12)	O1—C12—C13	116.36 (15)
C1—N1—C2	103.73 (15)	C11—C12—C13	121.01 (17)
C1—N1—C3	113.36 (14)	C14—C13—C12	120.94 (16)
C2—N1—C3	114.98 (14)	C14—C13—H13	119.5
N1^i^—C1—N1	109.0 (2)	C12—C13—H13	119.5
N1^i^—C1—H1A	109.9	C13—C14—C15	120.60 (17)
N1—C1—H1A	109.9	C13—C14—H14	119.7
N1^i^—C1—H1B	109.9	C15—C14—H14	119.7
N1—C1—H1B	109.9	C16—C15—C14	121.46 (18)
H1A—C1—H1B	108.3	C16—C15—C20	119.72 (18)
N1—C2—C2^i^	102.34 (10)	C14—C15—C20	118.82 (18)
N1—C2—H2A	111.3	C17—C16—C15	120.9 (2)
C2^i^—C2—H2A	111.3	C17—C16—H16	119.5
N1—C2—H2B	111.3	C15—C16—H16	119.5
C2^i^—C2—H2B	111.3	C16—C17—C18	119.7 (2)
H2A—C2—H2B	109.2	C16—C17—H17	120.1
N1—C3—C11	114.13 (14)	C18—C17—H17	120.1
N1—C3—H3A	108.7	C19—C18—C17	121.1 (2)
C11—C3—H3A	108.7	C19—C18—H18	119.5
N1—C3—H3B	108.7	C17—C18—H18	119.5
C11—C3—H3B	108.7	C18—C19—C20	121.08 (19)
H3A—C3—H3B	107.6	C18—C19—H19	119.5
C12—C11—C20	118.43 (16)	C20—C19—H19	119.5
C12—C11—C3	121.27 (17)	C15—C20—C11	120.07 (16)
C20—C11—C3	120.22 (15)	C15—C20—C19	117.41 (18)
O1—C12—C11	122.62 (16)	C11—C20—C19	122.50 (17)
			
C2—N1—C1—N1^i^	13.70 (8)	C13—C14—C15—C20	−1.4 (3)
C3—N1—C1—N1^i^	139.08 (14)	C14—C15—C16—C17	−178.1 (2)
C1—N1—C2—C2^i^	−34.89 (17)	C20—C15—C16—C17	1.6 (3)
C3—N1—C2—C2^i^	−159.24 (14)	C15—C16—C17—C18	0.2 (4)
C1—N1—C3—C11	166.25 (14)	C16—C17—C18—C19	−1.2 (4)
C2—N1—C3—C11	−74.64 (18)	C17—C18—C19—C20	0.4 (3)
N1—C3—C11—C12	−26.7 (2)	C16—C15—C20—C11	179.33 (16)
N1—C3—C11—C20	156.64 (15)	C14—C15—C20—C11	−1.0 (3)
C20—C11—C12—O1	175.45 (15)	C16—C15—C20—C19	−2.2 (3)
C3—C11—C12—O1	−1.3 (3)	C14—C15—C20—C19	177.39 (17)
C20—C11—C12—C13	−3.9 (3)	C12—C11—C20—C15	3.6 (3)
C3—C11—C12—C13	179.33 (16)	C3—C11—C20—C15	−179.57 (16)
O1—C12—C13—C14	−177.87 (16)	C12—C11—C20—C19	−174.69 (17)
C11—C12—C13—C14	1.5 (3)	C3—C11—C20—C19	2.1 (3)
C12—C13—C14—C15	1.2 (3)	C18—C19—C20—C15	1.3 (3)
C13—C14—C15—C16	178.20 (18)	C18—C19—C20—C11	179.66 (18)

Symmetry code: (i) −*x*+1, *y*, −*z*+1/2.

### Hydrogen-bond geometry (Å, º)

**Table:**

*D*—H···*A*	*D*—H	H···*A*	*D*···*A*	*D*—H···*A*
O1—H1···N1	1.05 (2)	1.65 (2)	2.6143 (19)	151.0 (19)
C2—H2*A*···O1^ii^	0.99	2.64	3.257 (2)	121

Symmetry code: (ii) *x*, −*y*+2, *z*−1/2.
